# Membrane‐Bound Multimodal Plasmonic Transducers for Noninvasive, In Situ Monitoring and Control of CAR T Cells Against Heterogeneous Solid Tumors

**DOI:** 10.1002/adma.74686

**Published:** 2026-08-19

**Authors:** Myeongsoo Kim, Ali Zamat, Melissa Cadena, Chloé Thiveaud, Riya Sen, Jeungyoon Lee, Elif Kulaksizoglu, S. Abbey Oliver, Don VanderLaan, Sydney Fábrega, Zizhen Zha, Kelsey P. Kubelick, Jinhwan Kim, Gabriel A. Kwong, Stanislav Y. Emelianov

**Affiliations:** ^1^ Petit Institute for Bioengineering and Bioscience Georgia Institute of Technology Atlanta Georgia USA; ^2^ Wallace H. Coulter Department of Biomedical Engineering Georgia Institute of Technology and Emory University School of Medicine Atlanta Georgia USA; ^3^ School of Electrical and Computer Engineering Georgia Institute of Technology Atlanta Georgia USA; ^4^ Department of Biomedical Engineering University of Virginia Charlottesville Virginia USA; ^5^ Department of Biomedical Engineering University of California Davis Davis California USA; ^6^ Department of Surgery School of Medicine University of California Davis Sacramento California USA; ^7^ Institute for Matter and Systems Georgia Institute of Technology Atlanta Georgia USA; ^8^ Cancer Technology Innovation Center Georgia Institute of Technology Atlanta Georgia USA; ^9^ Georgia Immunoengineering Consortium Emory University and Georgia Institute of Technology Atlanta Georgia USA; ^10^ Winship Cancer Institute Emory University Atlanta Georgia USA

**Keywords:** biomedical engineering, car t cells, multimodality, photoacoustic imaging in biomedicine, plasmonic nanostructures

## Abstract

Noninvasive monitoring and control of CAR T cells against heterogeneous solid tumors remain major challenges in understanding and improving treatment response. To address this, membrane‐bound plasmonic transducers, composed of plasmonically coupled gold nanospheres in an anisotropic framework, were developed for multimodal photoacoustic imaging and localized thermal modulation of CAR T cell activity. These transducers exhibit approximately 90% absorption efficiency and photostability under laser fluences exceeding 20 mJ cm^−2^, delivering photoacoustic and thermal responses over multiple lasing cycles. Membrane‐bound transducers on CAR T cells thus enable photoacoustic and thermal responsiveness upon laser excitation without compromising key cellular functions. In heterogeneous HER2‐expressing breast tumor models, longitudinal photoacoustic imaging enabled prospective stratification of tumors based on early T cell trafficking, predicting responders versus nonresponders with high sensitivity and specificity. Moreover, transducer‐mediated thermal modulation of intratumoral CAR T cells engineered with thermogenetic circuits to secrete T cell engagers redirected cytotoxicity toward antigen‐negative tumors, overcoming antigen escape and consequently enhancing therapy. Taken together, we demonstrate a strategy to noninvasively monitor and control CAR T cells against heterogeneous solid tumors via membrane‐bound multimodal transducers.

## Graphical abstract

Membrane‐bound multimodal transducers enable longitudinal ultrasound‐guided photoacoustic monitoring and in situ thermal control of CAR T cells in heterogeneous solid tumors. Imaging correlates T cell trafficking with tumor response, distinguishing responders from nonresponders. Transducer‐mediated thermogenetic modulation of intratumoral CAR T cells redirects cytotoxicity toward antigen‐negative tumors, overcoming antigen escape and enhancing therapy.

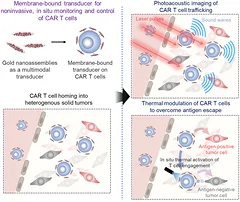

## 1 Introduction

Chimeric antigen receptor (CAR) T cell therapy leverages a patient's genetically engineered T lymphocyte to target tumor cells [1, 2, 3, 4]. While CAR T cell therapies have shown considerable success in treating hematological malignancies, therapeutic efficacy in patients with solid tumors has been inconsistent [1, 3]. One major challenge in treating solid tumors is antigenic heterogeneity, which regularly promotes antigen escape, allowing tumors to evade CAR‐mediated targeting [5, 6]. To address this limitation, recent efforts are focused on developing methods to monitor and bolster antitumor activities of CAR T cells within solid tumors by integrating either diagnostic [7, 8, 9, 10] or therapeutic [4, 11, 12, 13, 14, 15, 16, 17] nanoparticles (NPs). Examples include the use of imaging contrast agents for monitoring transferred CAR T cells, such as iron‐oxide NPs for magnetic resonance imaging [7, 8], or immunomodulators, such as multivalent antibody‐functionalized NPs or protein nanogels, that promote T cell engagement with tumor cells [12, 13, 14, 17]. Furthermore, to enhance control over the location and timing of CAR T cell activity, upconversion NPs have been combined with T cells engineered with an optogenetic circuit to conditionally activate T cell engagement [15]. However, most reported strategies have been evaluated in binary antigen‐positive or ‐negative tumor models, limiting their relevance to heterogeneous tumors, and primarily rely on unimodal NPs, which underscores the potential of multifunctional NPs that combine diagnostic and therapeutic functionalities to noninvasively monitor and enhance CAR T cell functions in situ against heterogeneous solid tumors.

The nonradiative conversion of electromagnetic waves into thermal energy by NPs has merit as a noninvasive tool for ultrasound‐guided photoacoustic (US/PA) imaging, which provides real‐time functional and anatomical information, alongside photothermal treatment that induces localized thermal deposition [18, 19, 20, 21, 22, 23, 24]. Across diverse NPs that serve as light‐to‐heat transducers, such as organic dyes [25, 26], iron‐oxide NPs [7], Prussian blue nanocubes [27, 28], and quantum dots [29], plasmonic gold nanoparticles (GNPs) are of particular interest due to the relatively large absorption cross‐section [18, 30], tunable optical spectrum [18, 30], physicochemical inertness [31], high thermal conductivity [19, 20], and versatile surface functionality [32]. Anisotropic GNPs, such as gold nanorods (GNRs), exhibit near‐infrared (NIR) light absorption correlated with their aspect ratio [18, 33, 34], enabling strong PA and thermal responses upon laser irradiation in the NIR optical window [19, 30]. However, their longitudinal utility is often compromised by reduced photostability as the photodamage threshold of GNPs is inversely proportional to their aspect ratio, leading to reshaping into gold nanospheres (GNSs) via atomic diffusion under laser excitation [35, 36, 37]. Therefore, achieving efficient light‐to‐heat conversion and photostability in GNPs could potentially leverage their multimodal functionalities for longitudinal diagnostic and therapeutic treatments over the course of CAR T cell therapy against solid tumors.

Here, we present a noninvasive platform for longitudinal monitoring and in situ enhancement of antitumor CAR T cell activities against heterogeneous solid tumors, enabled by cell membrane‐bound multimodal plasmonic transducers. These transducers are designed as gold nanoassemblies (GNAs), composed of plasmonically coupled GNSs within a nanosized anisotropic framework [38], demonstrating approximately 90% absorption efficiency at NIR wavelengths and photostability upon laser fluences exceeding 20 mJ cm^−2^. This characteristic offers longitudinal PA and thermal responses over multiple lasing cycles with laser parameters that damage conventional anisotropic GNPs. When conjugated to CAR T cell membrane, the membrane‐bound GNAs impart PA responses without altering key cellular functions, enabling longitudinal PA imaging of CAR T cell trafficking into solid tumors with heterogeneous antigen expression. This imaging reveals a correlation between CAR T cell trafficking and tumor response, allowing early differentiation of therapy responders from nonresponders with high specificity and sensitivity. Furthermore, by incorporating CAR T cells engineered with thermogenetic switches [39, 40, 41, 42] that trigger bispecific T cell engager (TCE) expression in response to transient mild hyperthermia (42°C), in situ thermal modulation of membrane‐bound GNAs allows local enhancement of antitumor activity to counter antigen escape, resulting in a significant improvement in therapeutic efficacy compared to conventional CAR T cells. Overall, longitudinal PA and thermal responses from membrane‐bound GNAs enable noninvasively monitoring and enhancing therapeutic efficacy of CAR T cells in situ in heterogeneous solid tumors.

### 1.1 GNAs Function as a Multimodal Photoacoustic and Thermal Transducer, Offering Longitudinal Signal Stability

The optical absorption of GNPs within the NIR optical window is directly correlated with their signal amplitude and contrast in PA imaging [43, 44, 45] and localized heat generation [18] in vivo. To ensure PA and thermal responses at NIR wavelengths, GNAs were fabricated as multimodal transducers by incorporating plasmonically coupled GNSs via a seed‐mediated growth within an anisotropic phenolic polymer space [38] (Figure 1a; Figure S1). The diameter and interparticle spacing of GNSs localized in the polymer space were precisely tuned by controlling the addition of gold ions during seed‐mediated growth. As the size of GNSs increased with closer interparticle spacing in GNAs, we observed an increase in absorption intensity at NIR wavelengths, due to enhanced interparticle plasmon coupling [46, 47, 48, 49]. This enhancement subsequently led to stronger PA responses and more efficient heat generation (Figure 1b–d; Figure S2a–g). The absorption efficiency of GNAs with close interparticle proximity was theoretically calculated to be approximately 90% using finite‐difference time‐domain (FDTD) simulation (Figure 1e; Figure S3), allowing for a distinguishable PA signal from GNAs down to a concentration of approximately 6 µg mL^−1^ (Figure 1f). Similarly, we observed a concentration‐dependent temperature increase by GNAs upon continuous‐wave (CW) laser irradiation (Figure 1g). Moreover, the photothermal conversion efficiency of GNAs was determined to be approximately 75% (Figure S4).

**FIGURE 1:**
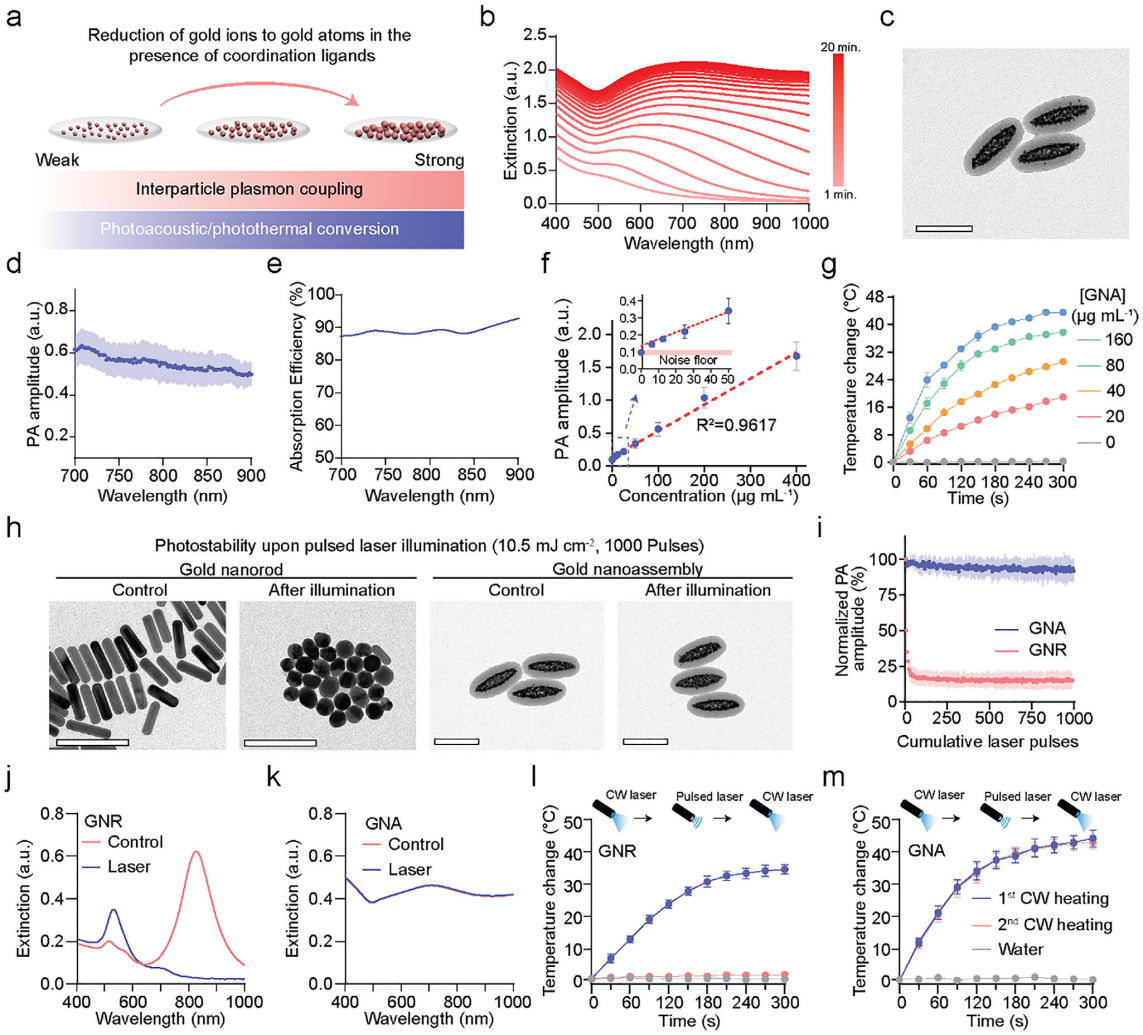
GNAs function as a multimodal photoacoustic and thermal transducer, offering longitudinal signal stability. a) Schematic illustration of a seeded growth strategy to fabricate GNAs consisting of anisotropically clustered GNSs. b) Real‐time UV‐vis‐NIR spectra of the synthesis of GNAs with a time interval of 1 min. c) TEM image of GNAs after the seeded growth. The scale bar is 200 nm. d) PA signal generation from the GNA solution within 700–900 nm spectral region (*n* = 7), mean ± s.d. e) Calculated absorption efficiency of GNA in the 700–900 nm spectral range. f) PA signal generation from the GNA solution with different mass concentrations at 800 nm (*n* = 4), mean ± s.d. g) Heat generation from GNA solution with different concentrations under 808 nm CW laser irradiation at 1 W cm^−2^ (*n* = 3), mean ± s.d. h) i) Structural stabilities (h) and PA responses (i) of GNRs or GNAs at a laser fluence of 10.5 mJ cm^−2^ (*n* = 3), mean ± s.d. j–m), UV‐vis‐NIR spectra (j,k) and repeatable CW laser‐mediated thermal responses (l,m) of GNRs or GNAs before and pulsed laser illumination at a laser fluence of 10.5 mJ cm^−2^ for 1,000 pulses (*n* = 3), mean ± s.d.

The photostability of GNAs is especially important when PA imaging and thermal modulation are applied across multiple sessions [19, 50]. Firstly, GNSs are photostable under pulsed laser illumination with fluence exceeding 50 mJ cm^−2^ [51]. Secondly, the polymer layer could improve structural stability under laser irradiation [52, 53]. Therefore, we tested the photostability of GNAs under pulsed and CW laser illumination, comparing their photostability with that of GNRs as a standard contrast agent [18, 30, 54]. Upon CW laser irradiation at 1 W cm^−2^ for 5 min, both GNAs and GNRs maintained their structural characteristics (Figure 1h; Figure S5). However, under pulsed laser irradiation at a laser fluence of 10.5 mJ cm^−2^ for 1,000 pulses, GNAs showed no changes in their structural and optical characteristics, producing stable PA signals, while GNRs became spheres and showed dramatic PA signal decay within 20 laser pulses (Figure 1h–k). In contrast to GNRs, the photostability of GNAs allowed for repeatable CW laser‐mediated heating cycles even after exposure to laser pulses (Figure 1l,m). Moreover, GNAs did not exhibit any significant changes in their optical characteristics under laser irradiation with a fluence of up to 20.2 mJ cm^−2^ (Figure S6). This photodamage threshold was higher compared to previously reported GNPs such as GNRs [55], silica‐coated GNRs [52], gold nanostars [50], and gold nanoplates [56] that are typically photodamaged at a laser fluence of 10 mJ cm^−2^. Furthermore, GNAs maintained their optical and colloidal stability over 20 days, suggesting their long‐term stability (Figure S7). Together, we demonstrate that GNAs can function as a multimodal imaging and thermal contrast agent, providing longitudinal signal stability across multiple cycles.

### 1.2 Membrane Conjugation of GNAs to T Cells Enhances NIR Light Absorption Without Compromising Key T Cell Functions

Since T cells produce negligible PA signals based on their lack of absorption in the NIR spectral range, engineering T cells with the ability to absorb light within the NIR window facilitates monitoring of their trafficking and infiltration into tumors through contrast‐enhanced PA imaging [9, 22]. We chemically conjugated GNAs to the free thiols on the membrane of T cells (Figure S8) by functionalizing maleimide groups [57, 58, 59] onto the surface of GNAs (Figure S9). The maleimide‐functionalized GNAs were incubated with wildtype primary human T cells (WT T cells; CD4:CD8 ratio = 1:2, Figure S10) at various GNA doses per cell to yield GNA‐bound WT T cells (GNA‐WT T cells), while removing unbound GNAs (Figure S11a–c). As the GNA conjugation dose per cell increased from 0 to 308 pg cell^−1^, the surface density of GNAs per cell area increased from 0 to 9.53 ± 4.26 µm^−2^, resulting in stronger optical absorption at NIR wavelengths (Figure 2a–c).

**FIGURE 2:**
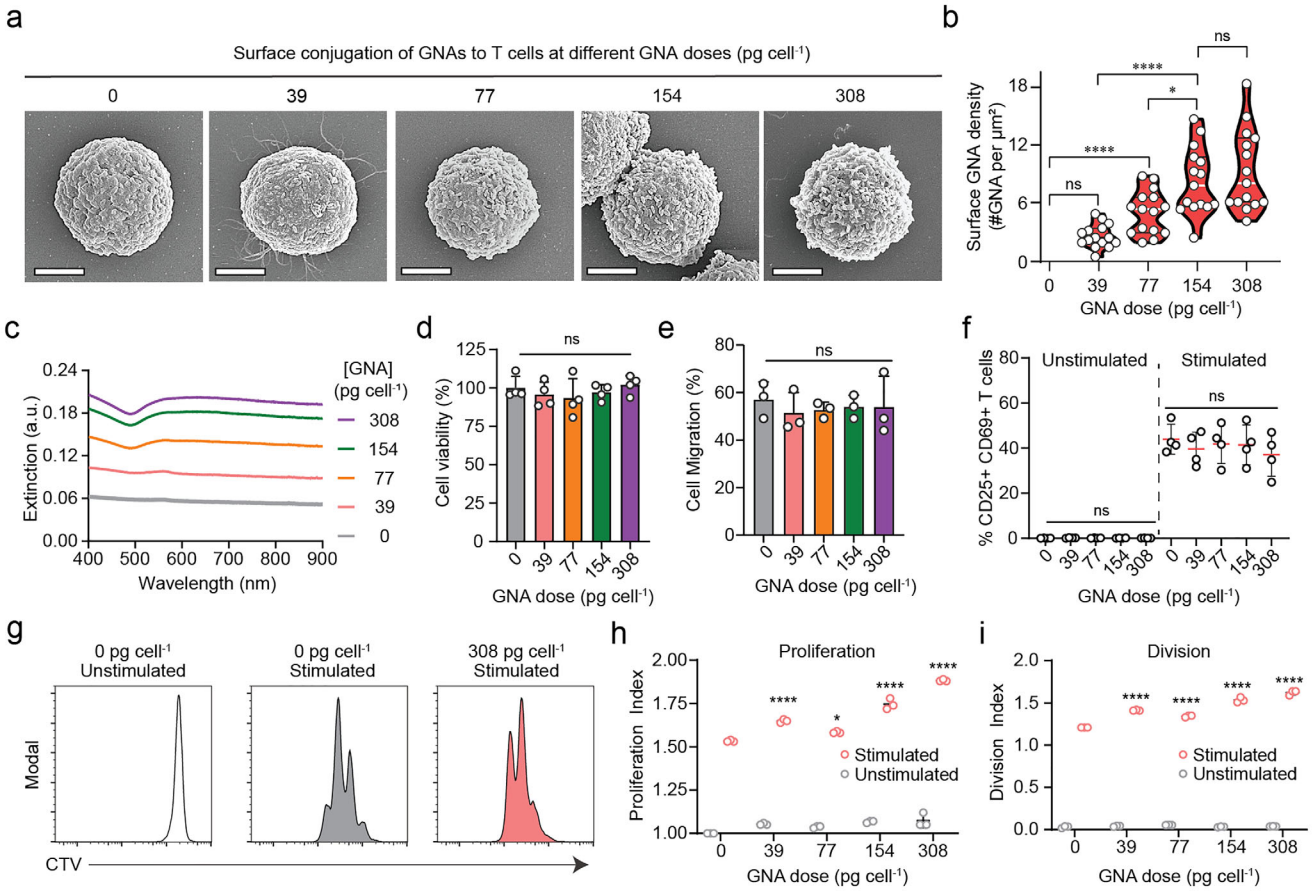
Membrane‐bound GNAs on T cells enable contrast‐enhanced PA imaging of T cells with longitudinal stability. a) Scanning electron microscope (SEM) imaging of GNA‐WT T cells following surface conjugation of GNAs at various doses. The scale bars are 2 µm. b) Quantification of surface density of GNAs per cell area (*n* = 15), mean ± s.d., ns = nonsignificant, ^*^ *p* < 0.05, ^****^ *p* < 0.0001; one‐way ANOVA with Tukey post‐hoc test. c) UV‐vis‐NIR spectra of GNA‐WT T cells coupled at different GNA doses. d) Cell viability 24 h post GNA conjugation at various doses (*n* = 4), mean ± s.e.m., ns = nonsignificant; one‐way ANOVA with Tukey post‐hoc test. e) Migration of GNA‐bound and unbound WT T cells (*n* = 3), mean ± s.e.m., ns = nonsignificant; one‐way ANOVA with Tukey post‐hoc test. f) Activation of GNA‐bound and unbound WT T cells following 48 h of dynabead stimulation as measured by CD25 and CD69 expression (*n* = 4), mean ± s.e.m., ns = nonsignificant; one‐way ANOVA with Tukey post‐hoc test. g–i), Flow cytometric analysis of GNA‐bound and unbound WT T cells 48 h post‐dynabead stimulation (g) and corresponding quantification of proliferation (h) and division (i) (*n* = 3), mean ± s.e.m, ^*^ *p* < 0.05, ^****^ *p* < 0.0001 signify comparisons of stimulated cohorts against 0 pg cell^−1^ condition; two‐way ANOVA with Tukey post‐hoc test.

We then investigated the effect of GNA conjugation on key cellular functions, including cell viability, proliferation, migration, and activation. The membrane conjugation of GNAs did not interfere with cell viability 24 h post‐labeling, irrespective of the surface density of GNAs per cell (Figure 2d). To examine T cell migration by chemotaxis, we performed migration assays and observed that the membrane‐bound GNAs unaltered the migration efficiency into lower chamber containing a chemoattractant, CXCL12 (Figure 2e; Figure S12). Furthermore, the membrane‐bound GNAs did not compromise T cell activation upon anti‐CD3/CD28 stimulation, displaying comparable CD25+CD69+ expression among all GNA doses evaluated (Figure 2f). Following activation assays, we next sought to evaluate the effect of the GNA conjugation on T cell proliferation by quantifying the dilution of cell trace violet (CTV) in the T cell populations (Figure 2g). We observed an increased proliferation (Figure 2h) and division (Figure 2i) index in GNA‐bound T cells at all doses tested (39 to 308 pg per cell) following CD3/CD28 stimulation as compared to unbound T cells. Moreover, the membrane‐bound GNAs on T cells remained on the surface of daughter cells following cell division, albeit with a decrease in surface density of GNAs per T cell (Figure S13). Taken together, these results demonstrate that the membrane‐bound GNAs on T cells enhance optical absorption without compromising key cellular functions.

### 1.3 Membrane‐Bound GNAs on T Cells Enable Contrast‐Enhanced PA Imaging of T Cells With Longitudinal Stability

Using US/PA imaging as a diagnostic tool for CAR T cell responses in solid tumors is facilitated by high imaging contrast over multiple imaging sessions. Therefore, a critical factor influencing the capability of GNA‐bound CAR T cells to monitor their spatial status in vivo lies in their imaging sensitivity and stability [19, 33, 43, 45]. Given that the imaging signal and contrast are associated with the intensity of optical absorption, we initially investigated the effect of the GNA dose in the cell membrane conjugation process on PA signal generation. Specifically, WT T cells conjugated with different GNA doses were embedded in a gelatin‐based tissue‐mimicking dome‐shaped phantom [60] at the same cell density for US/PA imaging (Figure 3a).

**FIGURE 3:**
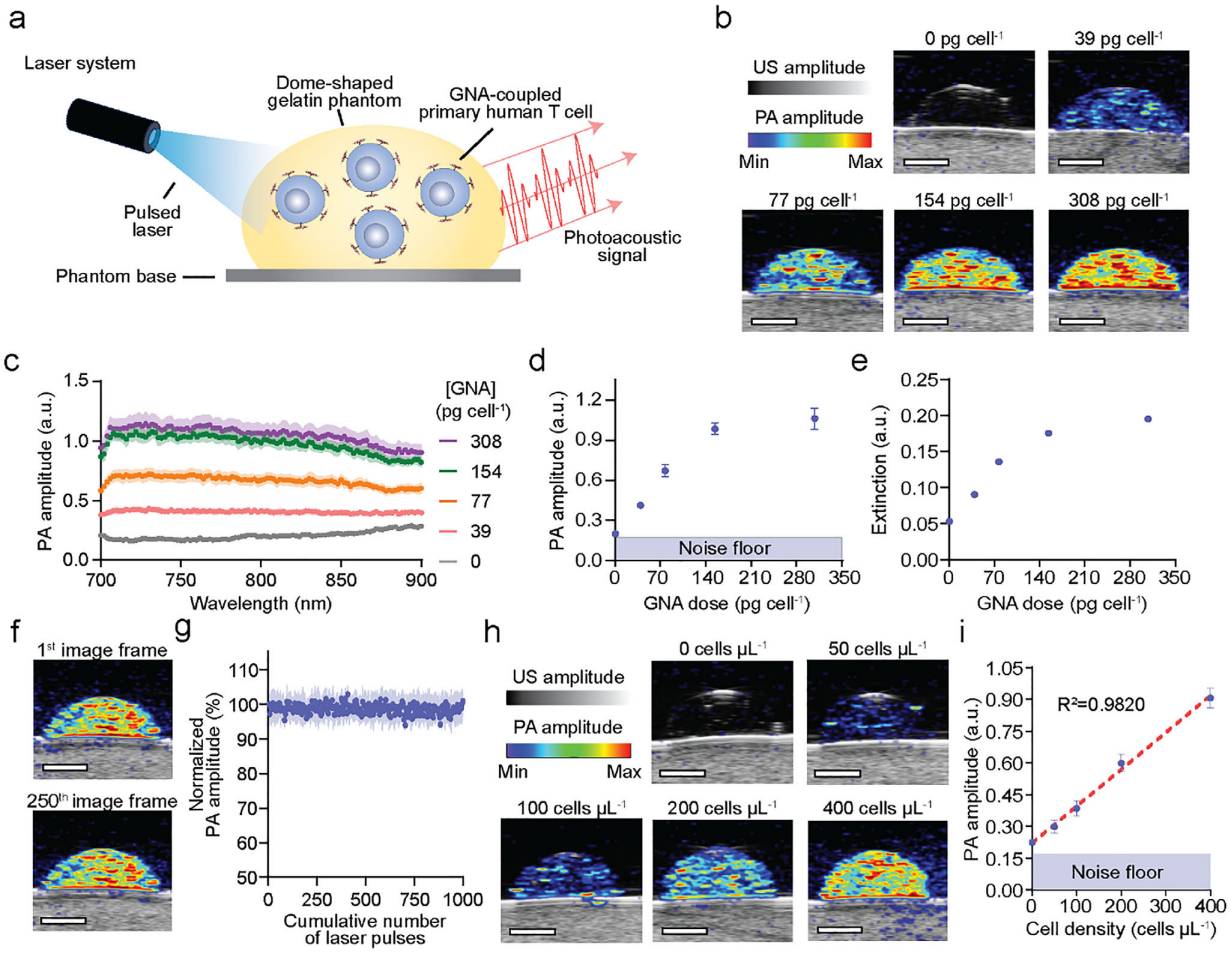
Membrane‐bound GNAs on T cells enable contrast‐enhanced PA imaging of T cells with longitudinal stability. a) Schematic illustration of in vitro US/PA imaging using a dome‐shaped tissue‐mimicking phantom containing GNA‐bound T cells b,c) US/PA images at 800 nm (b) and PA spectra (c) of the dome phantoms containing T cells conjugated at different doses of GNAs at an identical cell density of 400 cells µL^−1^ (*n* = 3), mean ± s.e.m. d,e) PA amplitude (d) and extinction intensity at 800 nm (e) of T cells conjugated at different GNA doses (*n* = 3), mean ± s.e.m. f) US/PA images of GNA‐bound T cells (GNA conjugation dose of 154 pg cell^−1^) at the first and 250^th^ imaging frames. g) PA signals form GNA‐bound T cells (at a conjugation dose of 154 pg cell^−1^) for 1,000 pulses at a laser fluence of 10.5 mJ cm^−2^ (*n* = 3), mean ± s.e.m. h,i) US/PA images (h) and PA amplitude (i) of the dome phantom containing different densities of GNA‐bound T cells (conjugation dose of 154 pg cell^−1^) at 800 nm (*n* = 3), mean ± s.e.m. The scale bars for imaging results are 2 mm.

As the GNA conjugation dose increased from 0 to 308 pg per cell, the PA imaging contrast increased at a wavelength of 800 nm in the dome phantoms containing cells at an identical density of 400 cells µL^−1^ (Figure 3b). Furthermore, owing to the intense optical response of membrane‐bound GNAs at broad NIR wavelengths, GNA conjugation process with a dose of 308 pg cell^−1^ led to an approximately fivefold increase in PA amplitude in the spectral range from 700 nm to 900 nm compared to unconjugated cells (Figure 3c; Figure S14a–c). PA signal enhancement is correlated with the intensity of optical absorption in cells conjugated with different GNA doses (Figure 3d,e).

Having established that the membrane conjugation of GNA to T cells at a dose of 154 pg cell^−1^ enables contrast‐enhanced PA imaging, we next tested the imaging stability and sensitivity of GNA‐bound T cells at this specific dose. The GNA‐bound T cells produced stable PA signals with no decay at a laser fluence of 10.5 mJ cm^−2^ over 1,000 laser pulses across 250 imaging frames (Figure 3f,g). GNA‐bound and unbound T cells exhibited comparable viability after pulsed laser irradiation at these parameters (Figure S15), suggesting no adverse impact of the PA conversion of the membrane‐bound GNAs on T cell viability. Moreover, GNA‐bound T cells were detectable at a density down to 50 cells µL^−1^ in the phantom, corresponding to a total of 1,500 cells (Figure 3h,i). Collectively, these in vitro imaging results demonstrate the capability of GNA‐bound T cells for US/PA imaging, offering high imaging contrast and longitudinal imaging stability.

### 1.4 Longitudinal PA Monitoring of GNA‐Bound CAR T Cells in Heterogeneous Solid Tumors Reveals a Correlation Between T Cell Trafficking and Tumor Response, Distinguishing Therapy Responders From Nonresponders

The trafficking of CAR T cells into solid tumors with heterogeneous antigen profiles is a major challenge in developing effective CAR T cell therapies against solid cancers [1, 2, 3] but has not been explicitly characterized. We therefore sought to leverage PA imaging capability of GNA‐bound CAR T cells to understand the impact of heterogeneous antigen expression on trafficking and subsequent therapeutic efficacy of CAR T cells. As the membrane conjugation of GNAs to T cells at a dose of 154 pg cell^−1^ enhances PA signals with a saturated GNA surface density, we first examined the effect of GNA conjugation at this dose on the cytotoxicity of CAR T cells against both CAR antigen‐negative and ‐positive tumors. αHER2 CAR T cells were conjugated with GNAs (GNA‐αHER2 CAR T cells) before coincubation with HER2+ or HER2‐ MDA‐MB‐468 cancer cells at an effector‐to‐target ratio of 2:1. We first observed that GNA‐WT T cells did not show off‐target toxicity on HER2‐ or HER2+ MDA‐MB‐468 tumor cells (Figure S16, left two panels). Furthermore, we observed that GNA‐αHER2 CAR T cells specifically killed HER2+ target tumor cells (Figure S16, right two panels). These findings suggest that the membrane‐bound GNAs on CAR T cells do not affect targeting and killing of antigen‐positive tumor cells without unintended cytotoxicity against antigen‐negative cells.

Breast tumors are typically categorized as HER2 positive when they exhibit HER2 expression levels of approximately 10% [61]. Treating a heterogeneous tumor with such low expression could be challenging, as it may lead to an inconsistent therapeutic response based on restricted ability in tumor homing and retention of CAR T cells. To investigate the effect of trafficking of GNA‐αHER2 CAR T cells into heterogeneous MDA‐MB‐468 tumors via longitudinal US/PA imaging (Figure 4a), NSG mice were inoculated with MDA‐MB‐468 with tumors comprising 0%, 10%, 50%, or 100% HER2+ expressing cells (Figure S17), followed by adoptive cell transfer (ACT) of GNA‐αHER2 CAR T cells. The tumors for each mouse were longitudinally monitored by US/PA imaging at a wavelength of 800 nm before and after ACT (Figure 4b–d; Figures S18 and S19). Before ACT, all tumors showed no significant difference in PA signal (Figure 4b,c). However, on day 4 post‐ACT, tumors with 50% and 100% HER2+ compositions exhibited higher PA signals than those with 0% and 10% HER2+ compositions (Figure 4b,c). Notably, increased PA amplitude was observed in 50% and 100% HER2+ tumors before tumor volumes diverged among the cohorts, indicating increased trafficking and infiltration of GNA‐αHER2 CAR T cells into the tumors compared to those in 0% and 10% HER2+ tumors (Figure 4c,d; Figures S18–20). Additionally, in 50% and 100% HER2+ tumors, PA signals were detected throughout the tumor, whereas in 0% and 10% HER2+ tumors, PA signal was primarily sequestered at the periphery (Figure 4b; Figure S18). Furthermore, on day 15 post‐ACT, a decrease in PA signals was observed in 50% and 100% HER2+ tumors, showing comparable PA signals with 0% and 10% HER2+ tumors (Figure 4c; Figure S19). This signal decrease may be due to the reduction in the density of membrane‐bound GNAs as tumor‐infiltrating CAR T cells proliferate, along with decrease in the number of GNA‐αHER2 CAR T cells throughout tumor regression (Figure 4d; Figure S20). Moreover, early increases in tumor PA signal amplitudes from day −1 to day 4 post‐ACT were anti‐correlated with tumor volume changes observed between day −1 and day 21 post‐ACT, during which marked differences in tumor growth or regression were evident (R = −0.8103, p < 0.0001) (Figure 4e).

**FIGURE 4:**
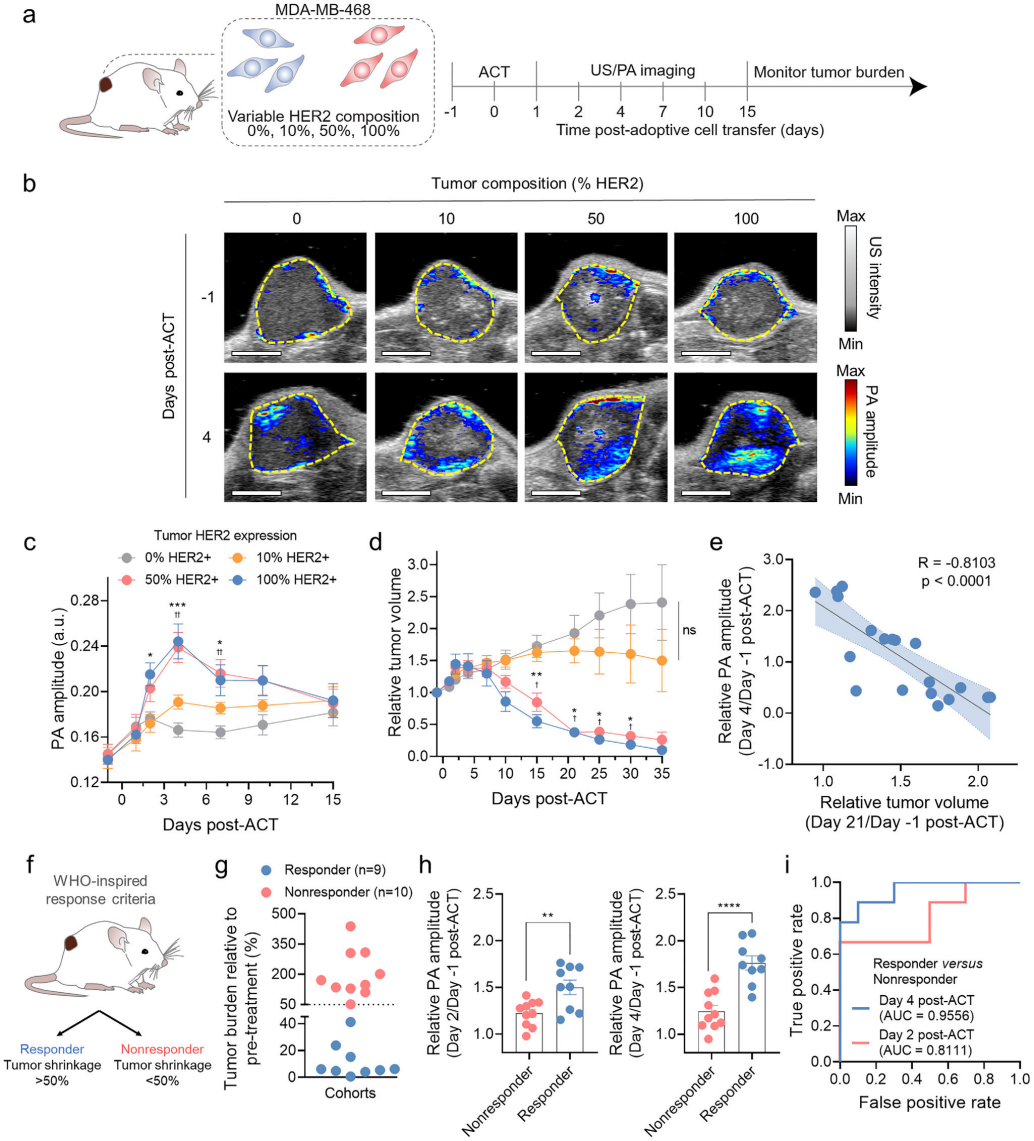
Longitudinal PA monitoring of GNA‐bound CAR T cells in heterogeneous solid tumors reveals a correlation between T cell trafficking and tumor response, distinguishing therapy responders from nonresponders. a) Schematic representation of timeline for in vivo US/PA monitoring of trafficking of GNA‐αHER2 CAR T cells into MDA‐MB‐468 tumors with varying HER2 expression. b) US/PA imaging (800 nm) of tumors at indicated time points post‐ACT. The scale bars are 4 mm. c) Corresponding PA amplitudes of tumors at 800 nm (*n* = 5), mean ± s.e.m., ^*,†^P < 0.05, ^**,††^P < 0.01, ^***^P < 0.001 (^*^; HER2 100% vs. HER2 0%, ^†^;HER2 50% vs. HER2 0%); one‐way ANOVA with Tukey post‐hoc test. d) Relative tumor volumes (*n* = 4 to 5), mean ± s.e.m., ns = nonsignificant, ^*,†^P < 0.05, ^**^P < 0.01 (^*^; HER2 100% vs. HER2 0%, ^†^;HER2 50% vs. HER2 0%); mixed‐effects analysis with Tukey post‐hoc test. e) Correlation analysis between relative tumor volume (day 21/day −1 post‐ACT) and relative PA amplitude (day 4/day −1 post‐ACT). The linear regression model was fitted to the data with 95% confidence band. f,g) Schematic illustration of the classification of responders and nonresponders based on WHO‐inspired response criteria in ACT‐treated mice bearing tumors with varying HER2 expression (f) and corresponding relative changes in tumor burden compared with Day 1 post‐ACT (g, *n* = 19). h) Early PA signal changes (day 2/day−1 post‐ACT or day 4/day −1 post‐ACT) in responder (*n* = 9) and nonresponder groups (*n* = 10), mean ± s.e.m., ^***^ *p* < 0.001; two‐tailed student's t‐test. i) Receiver operating characteristic (ROC) curves showing the ability of early T cell trafficking‐associated PA imaging signals to distinguish responders from nonresponders following ACT.

Next, we evaluated the ability of membrane‐bound GNAs to distinguish ACT responders from nonresponders based on early PA signal elevations in tumors with varying HER2 expression (Figure 4f). Tumor responses were evaluated by longitudinal US measurements of tumor volumes using World Health Organization (WHO)‐inspired response criteria [62, 63] based on percent reduction from baseline. Changes in tumor burden were calculated relative to baseline measurements obtained on Day −1 post‐ACT, that is, prior to treatment initiation. Mice were classified as responders if they exhibited ≥50% reduction in tumor volume and as nonresponders if they showed <50% reduction or tumor growth over the 35‐day treatment period (Figure 4g; Figure S21). Tumors in the responder group displayed significantly higher PA amplitude elevations on Days 2 and 4 post‐ACT compared to nonresponders (Figure 4h). Receiver operating characteristic (ROC) curve analysis of early PA signal changes yielded areas under the curve (AUCs) of 0.8111 and 0.9556 on Days 2 and 4 post‐ACT, respectively, demonstrating that membrane‐bound GNAs can robustly stratify ACT responders from nonresponders with high sensitivity and specificity (Figure 4i). Consistent with the early PA signal elevations observed in responder tumors, immunohistochemical and flow cytometric analysis on Day 4 post‐ACT demonstrated increased T cell infiltration (Figure S22) and a higher frequency of CD25+CD69+ activated T cells in responder tumors compared with nonresponder tumors (Figure S23), further validating that early PA signals reflect CAR T cell accumulation and activation within the tumor microenvironment. Together, these findings highlight the potential of longitudinal PA imaging of GNA‐bound CAR T cells to provide early insight into therapeutic efficacy in heterogeneous solid tumors before detectable changes in tumor growth occur.

### 1.5 In Situ Thermal Control of GNA‐Bound Thermogenetic CAR T Cells Overcomes Antigen Escape in Heterogeneous Solid Tumors

Differential expression of tumor antigens [6] can lead to evasion of CAR T cell‐mediated targeting [64]. Therefore, we sought to explore whether combining heat‐mediated expression of a T cell engager (TCE) targeting NKG2D ligands, present on approximately 90% of human tumors [65, 66], with membrane‐bound GNAs could address the challenge of tumor antigen escape in heterogeneous solid tumors by redirecting CAR T cell cytotoxicity toward antigen‐negative tumor cells.

We previously designed a thermogenetic switch (TS) [67, 68] capable of driving transgene expression in CAR T cells [39, 41] following mild hyperthermia treatment at 42°C (Figure 5a). First, we evaluated the impact of membrane conjugation of GNAs on TS activation and observed comparable transgene activation of a *Gaussia* luciferase (Gluc) reporter following thermocycler‐mediated heating at 42°C for 20 min between GNA‐bound TS‐Gluc T cells (GNA‐TS‐Gluc T cells) and unbound cells (Figure S24). This result suggests no adverse impact of the membrane‐bound GNAs on the TS activation. Subsequently, we sought to evaluate the effect of GNA‐mediated heating of GNA‐TS‐Gluc T cells on the transgene activation. Under controlled CW laser illumination, GNA‐TS‐Gluc T cells exhibited a rapid temperature increase to 42°C within 5 min and maintained stable temperature plateau for 20 min whereas TS‐Gluc cells remained at room temperature (Figure 5b) without affecting viability (Figure S25). Consequently, Gluc expression was approximately 15‐fold higher in GNA‐TS‐Gluc T cells when compared to unconjugated cells which had nonsignificant signal relative to unheated cells (Figure 5c,d). Moreover, under pulsed laser illumination, both GNA‐bound and unbound cells had undetectable Gluc expression (Figure 5c,d), indicating that the genetic switch in CAR T cells is not activated by the US/PA imaging sessions and further confirming the specificity of CW laser‐mediated thermal actuation of membrane‐bound GNAs for thermogenetic activation.

**FIGURE 5:**
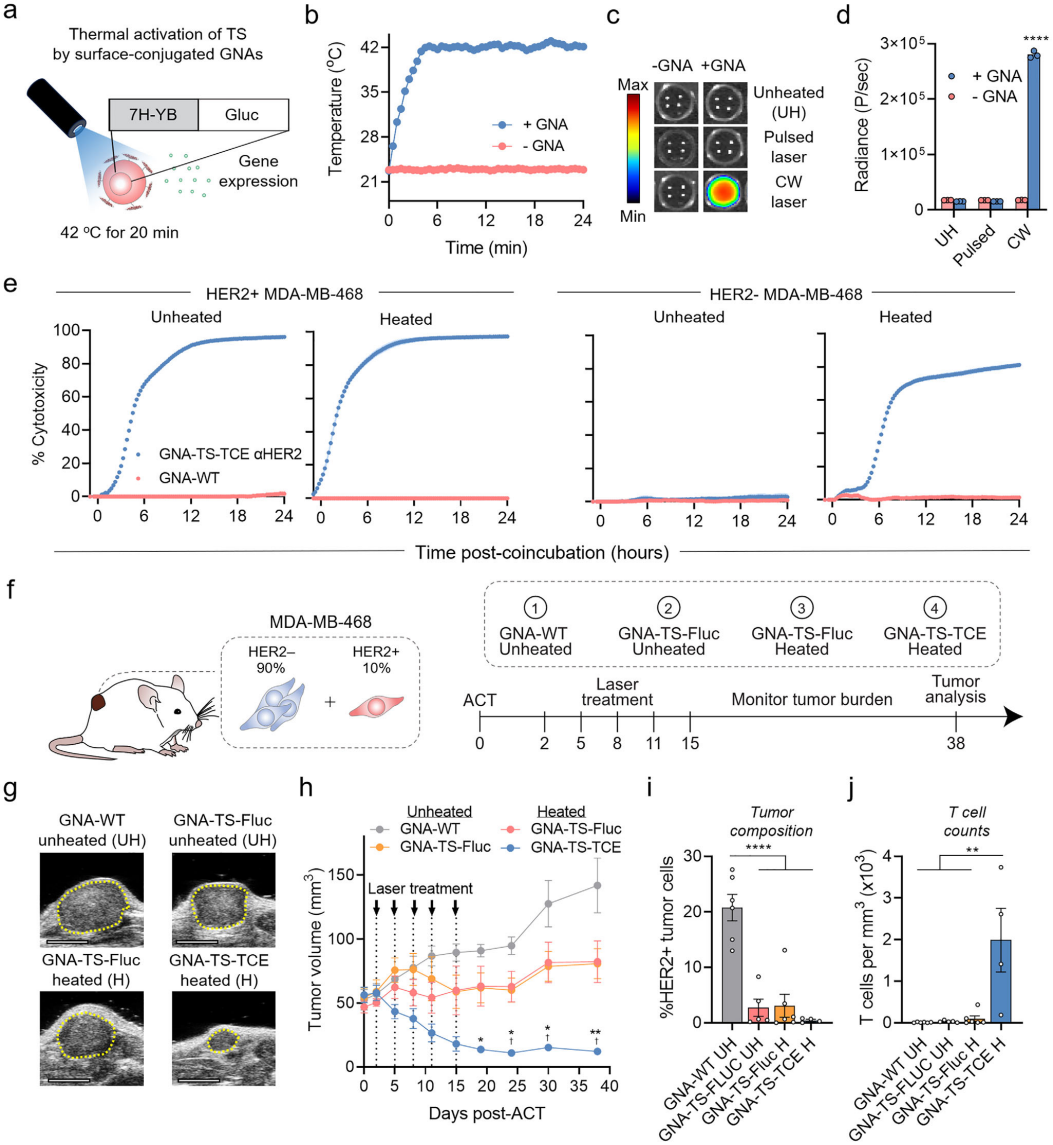
In situ thermal control of GNA‐bound thermogenetic CAR T cells overcomes antigen escape in heterogeneous solid tumors. a) Schematic representation of thermal activation of GNA‐TS engineered CAR T cells to express transgenes. b) Temperature profiles of GNA‐TS Gluc T cells and unbound Gluc T cells under 808 nm‐CW laser irradiations at a controlled laser intensity (*n* = 3), mean ± s.e.m. c) Luminescence images of wells containing GNA‐TS‐Gluc T cells and unbound TS‐Gluc T cells after irradiation with the CW or pulsed laser light. d) Corresponding quantification of Gluc expression (*n* = 3), mean ± s.e.m., ^****^ *p* < 0.0001; one‐way ANOVA with Tukey post‐hoc test. e) Cytotoxicity of GNA‐WT or GNA‐TS‐TCE αHER2 CAR T cells. Cells were unheated or heated to 42 °C prior to coincubation and quantification of cytotoxicity against HER2‐ or HER2+ MDA‐MB‐468. f) Schematic representation of timeline for in situ thermal modulation of CAR T cells localized in heterogeneous MDA‐MB‐468 tumors. g) Representative US tumor images at day 30 post‐ACT. The scale bars are 4 mm. h) Quantification of tumor burden following ACT (*n* = 5 to 6), mean ± s.e.m., ^*,†^P < 0.05, ^**^P < 0.01 (^*^; TS‐TCE heated vs. TS‐Fluc unheated, ^†^; TS‐TCE heated vs. TS‐Fluc unheated); mixed‐effects analysis with Tukey post‐hoc test. i) tumor composition and j) T cell counts 38 days post‐ACT (*n* = 4 to 6), mean ± s.e.m., ^**^ *p* < 0.01, ^****^ *p* < 0.0001; one‐way ANOVA with Tukey post‐hoc test.

We then explored the potential of thermal modulation by membrane‐bound GNAs to mediate NKG2D‐targeting TCE expression in thermogenetic CAR T cells (Figure 5e). We first confirmed the heat‐inducible TCE expression in TS‐TCE CAR T cells following thermal stimulation (Figure S26). Next, we evaluated cytotoxicity of WT or TS‐TCE αHER2 CAR T cells conjugated with GNAs, against HER2+ or HER2‐ MDA‐MB‐468 cells which express NKG2DL [39]. When coincubated with HER2+ tumor cells, GNA‐bound TS‐TCE αHER2 CAR T (GNA‐TS‐TCE CAR T) cells exhibited comparable cytotoxicity profiles with or without heat treatment at 42°C for 20 min, suggesting that thermal treatment does not impair αHER2 CAR‐mediated targeting. By contrast, against HER2‐ cancer cells, neither GNA‐WT nor GNA‐TS‐TCE αHER2 CAR T cells exhibited cytotoxicity in the absence of heating (Figure 5e). However, following heat treatment at 42°C for 20 min, a significant cytotoxic activity was observed against HER2‐ cancer cells by GNA‐TS‐TCE αHER2 CAR T cells compared to GNA‐WT T cells (Figure 5e), supporting that membrane‐bound GNAs enable in situ thermal activation of TCEs without compromising antigen specificity.

To evaluate whether this strategy could mitigate heterogeneous antigen escape through localized thermal modulation in vivo, we first assessed the CW laser‐mediated photothermal heating capability of GNAs using chicken breast tissue as a tissue‐mimicking barrier. GNAs maintained efficient light‐to‐heat conversion and generated an approximately 10 °C temperature increase following laser irradiation through 10 mm of chicken breast tissue (Figure S27). Furthermore, we validated that repeated heat treatment within the mild hyperthermia regimen (42°C for 20 min every 48 h for three consecutive treatments) did not impair T cell viability, proliferation, or CD69 expression (Figure S28). We further confirmed that GNA‐WT T cells with laser treatments did not lead to tumor regression (Figure S29). We then inoculated NSG mice with ~10% HER2+ MDA‐MB‐468 tumors (Figure S30) and intravenously administered GNA‐TS‐TCE αHER2 CAR, GNA‐TS‐Fluc αHER2 CAR, or GNA‐WT T cells (Figure 5f). CW laser treatment to maintain tumor temperature at 42°C for 20 min was then carried out every 3–4 days following ACT (days 2, 5, 8, 11, and 15). Compared to GNA‐WT control mice which did not receive CAR T cells or laser treatments, administration of GNA‐TS‐Fluc αHER2 CAR with or without laser irradiation did not lead to a reduction in tumor burden. By contrast, cohorts which received both GNA‐TS‐TCE αHER2 CAR T cells and laser treatments markedly reduced tumor burden for the duration of the study (Figure 5g,h; Figure S31). To determine whether antigen escape contributed to the lack of tumor clearance, we assessed HER2 expression within remaining tumors. Compared to GNA‐WT control cohorts which maintained expression of HER2 on the surface of tumor cells (20.7%), mice treated with αHER2 CAR T cells had a significant decrease in the frequency of HER2+ tumor cells ranging from 0.4% to 3% (Figure 5i), indicating that the resultant tumor volume is attributed to tumor antigen escape from CAR T cell targeting. Furthermore, quantification of HER2‐negative tumor cells demonstrated that GNA‐TS‐TCE αHER2 CAR T cells with laser treatments significantly reduced the abundance of antigen‐negative tumor populations compared with other groups (Figure S32), confirming the ability of heat‐induced TCE expression to redirect killing activity against HER2‐negative tumor cells in vivo. Flow cytometric analysis of T cells within the tumor revealed that cohorts treated with GNA‐TS‐TCE αHER2 CAR T cells had an increased density when compared to GNA‐WT or GNA‐TS‐Fluc αHER2 CAR T controls (Figure 5j). Collectively, these results confirm that heat‐mediated local expression of TCEs, facilitated by membrane‐bound GNAs under image guidance, could ultimately enhance CAR T cell therapy in heterogeneous solid tumors.

## 2 Discussion

This work establishes a strategy to enhance noninvasive monitoring and control of CAR T cells against heterogeneous solid tumors via membrane‐bound GNAs as multimodal light‐to‐heat transducers. To achieve this, we first demonstrated the plasmon coupling‐induced optical response of GNAs at NIR wavelengths and their longitudinal stability under both pulsed and CW laser irradiation, ensuring effective PA responses and thermal modulation over multiple imaging and heating sessions. GNAs offer several advantages over anisotropic GNPs currently used as a light‐to‐heat transducer, such as GNRs, for both PA imaging and photothermal applications. Firstly, GNAs efficiently absorb incident light with a high absorption efficiency of around 90% across a broad range of NIR wavelengths, leading to effective PA and thermal responses throughout the entire NIR optical window. Theoretically, the absorption efficiency is inversely proportional to the volume of GNPs due to the increased contribution of scattering to optical responses [18]. For instance, when GNRs exceed a dimension of 100 nm, their absorption efficiency drops below 70% [18, 50]. By contrast, we found that assembling small GNSs in proximity resulted in absorption dominance in optical response across the broad NIR spectral range. This approach provides a strategy for designing GNPs that accomplishes efficient light‐to‐heat conversion for multimodal imaging and therapeutic applications. Secondly, unlike traditional anisotropic GNPs, including GNRs [55], silica‐coated GNRs [52], gold nanostars [50], and gold nanoplates [56], GNAs exhibit a photodamage threshold over a laser fluence of 20 mJ cm^−2^ under laser irradiation. This superior photostability enables longitudinal diagnostic and therapeutic applications.

Since CAR T cells produce negligible optical absorption in the NIR window, we performed membrane conjugation of GNAs to CAR T cells for contrast‐enhanced PA imaging. In vitro characterizations confirmed that the membrane‐bound GNAs do not interfere with any key cellular functions, including viability, activation, proliferation, migration, and cytotoxicity against target cancer cells. Although membrane‐bound GNAs did not induce spontaneous T cell activation, we observed a modest increase in proliferation under TCR stimulation conditions, indicating that membrane‐bound GNAs may influence activation‐induced T cell responses. Future studies investigating the underlying molecular mechanisms will further clarify the interactions between membrane‐conjugated nanomaterials and T cell signaling pathways. Building on these findings, we demonstrated that US/PA imaging of GNA‐bound CAR T cells was used to monitor their trafficking into MDA‐MB‐468 tumors with heterogeneous HER2 expressions, ranging from 0% to 100%. We found that treatment with GNA‐αHER2 CAR T cells that targeted a single antigen decreased tumor burden of 50% and 100% HER2‐expressing tumors, with an observed increase in PA signal amplitudes up to day 4 post‐ACT. By contrast, CAR T cells targeting 10% HER2‐expressing tumors exhibited nonsignificant therapeutic efficacy compared to 0% HER2‐expressing tumors. Nonetheless, we observed PA signal increases for both 0% and 10% HER2 tumors compared to before ACT, suggesting that transferred CAR T cells localized into the tumor region even with low CAR antigen expression. Furthermore, it is noteworthy that PA imaging following ACT revealed time‐dependent PA signal profiles in each tumor, corresponding to different antigen compositions by day 4 post‐ACT, despite there being no significant differences in tumor volumes across the various antigen levels. Moreover, longitudinal PA imaging of GNA‐bound CAR T cells in tumors with heterogeneous antigen expression revealed a significant correlation between T cell trafficking and tumor response, enabling robust stratification of therapy responders versus nonresponders. Changes in GNA‐associated imaging signals due to T cell proliferation, GNA redistribution to daughter cells, surface internalization, cell–cell transfer, or decreased T cell density during tumor regression, may limit the utility of membrane‐bound GNAs for long‐term T cell tracking. Nevertheless, early imaging‐based assessment of CAR T cell trafficking and infiltration, prior to substantial signal dilution, remains a valuable indicator of therapeutic efficacy in heterogeneous solid tumors.

Lastly, we demonstrated that the localized, in situ thermal modulation of GNA‐bound CAR T cells with a thermogenetic circuit enhanced antitumor activity toward 10% HER2‐expressing MDA‐MB‐468 tumors by redirecting cytotoxicity of CAR T cells toward antigen‐negative tumors through the expression of NKG2DL‐targeting TCE. We observed the negligible TS‐driven transgene expression upon pulsed laser irradiation, indicating that light‐to‐heat conversion of the membrane‐bound GNAs under nanosecond‐pulsed laser irradiation could not reach the temperature threshold required for the TS activation. This outcome allowed us to use the US/PA imaging modality as a tool to guide trajectories relative to anatomy for local thermal modulation, without the concern of inadvertent TS activation during imaging sessions. Although localized mild hyperthermia may induce transient cellular stress responses or inflammatory signaling within the tumor microenvironment, our control experiments demonstrated that thermal stimulation alone was insufficient to induce tumor regression. These findings suggest that the enhanced antitumor activity observed with GNA‐TS‐TCE αHER2 CAR T cells primarily results from heat‐induced TCE expression and subsequent redirection of CAR T cell cytotoxicity, rather than nonspecific effects associated with thermal stimulation. Therefore, we envision that the current abilities to noninvasively monitor and modulate cellular functions can be expanded to other cellular immunotherapies, not limiting to CAR T cell therapies alone.

Together, we demonstrate that membrane‐bound GNAs serve as multimodal transducers for noninvasive imaging and in situ control of CAR T cells in the treatment of heterogeneous solid tumors. This approach has been used to study the impact of CAR T cell trafficking on therapeutic efficacy against solid tumors with heterogeneous antigen profiles by longitudinal US/PA imaging, indicating a correlation between early PA signals and tumor responses. Introducing a thermogenetic circuit, designed to trigger TCE expression in response to mild hyperthermia, further empowers GNA‐bound CAR T cells to locally enhance antitumor responses upon laser‐mediated thermal modulation by membrane‐bound GNAs under image guidance, overcoming hurdles associated with tumor antigen escape. Overall, our approach highlights the dual utility of membrane‐bound GNAs for both monitoring and augmenting CAR T cell therapies in solid tumors with heterogeneous antigen expression.

## 3 Experimental Section

Experimental details, including the fabrication, characterization, and numerical analysis of GNAs, conjugation of GNAs to the T cell membrane, characterization of GNA‐bound T cells, and in vivo imaging and therapeutic protocols, can be found in Supplementary Note 1 of the Supporting Information.

### 3.1 Statistical Analysis

Statistical analysis was performed using GraphPad Prism 8.0.2 statistical software. Flow cytometry data were collected with Aurora (Cytek) and analyzed using FlowJo X (FlowJo). All acquired US/PA imaging data were analyzed using VevoLAB 5.7.0 and MATLAB software. In vitro IVIS data were collected and analyzed with Living Image 4.4.5 (PerkinElmer). Figures were designed in Adobe Illustrator. No data transformation or normalization was applied unless otherwise specified. Data are presented as mean ± s.d. or mean ± s.e.m. as indicated in the corresponding figure legends. Sample sizes (n) and the statistical tests used for each comparison are provided in the corresponding figure legends. Different numbers of asterisks were assigned to the graphs showing the statistical comparison across different groups (ns: nonsignificant difference; ^†^or ^*^: *p* < 0.05; ^††^ or ^**^: *p* < 0.01, ^***^: *p* < 0.001, ^****^: *p* < 0.0001).

## Author Contributions

M.K., A.Z., G.A.K., and S.Y.E. conceived and designed the project. M.K., A.Z., M.C., C.T., R.S., J.L., E.K., A.O., D.V., and Z.Z. performed the experiments. M.K., A.Z., M.C., C.T., K.P.K., and J.K. analyzed the data. M.K., A.Z., M.C., C.T., G.A.K., and S.Y.E. interpreted results. K.P.K. and J.K. provided technical input by conducting earlier foundational studies that helped to design and guide the current imaging and GNA surface conjugation experiments. M.K., A.Z., G.A.K., and S.Y.E. wrote the draft of the manuscript. All authors discussed and commented on the manuscript.

## Conflicts of Interest

G.A.K. reports equity or consulting roles for Port Therapeutics, Send Biotherapeutics, and Ridge Biotechnologies. The terms of this arrangement have been reviewed and approved by Georgia Tech in accordance with its conflict‐of‐interest policies.

## Supporting information




**Supporting File**: adma74686‐sup‐0001‐SuppMat.docx.

## Data Availability

The data and code that support the findings of this study within the paper and the Supplementary Information are available from the corresponding authors upon reasonable request.
