# Supplementary material for: Membrane‐Bound Multimodal Plasmonic Transducers for Noninvasive, In Situ Monitoring and Control of CAR T Cells Against Heterogeneous Solid Tumors
Source: Adv Mater. 2026 Aug 19;38(58):e74686. doi: 10.1002/adma.74686 (PMC13674615; doi:10.1002/adma.74686)
Supplement: Supplementary file 1 [file ADMA-38-e74686-s001.docx]

**Supplementary Information**

Membrane-bound multimodal plasmonic transducers for noninvasive, in situ monitoring and control of CAR T cells against heterogeneous solid tumors

Myeongsoo Kim^1,2,3,11^, Ali Zamat^2,11^, Melissa Cadena^2^, Chloé Thiveaud^2^, Riya Sen^2^, Jeungyoon Lee^2,3^, Elif Kulaksizoglu^2^, S. Abbey Oliver^2^, Don VanderLaan^2,3^, Sydney Fábrega^2^, Zizhen Zha^2^, Kelsey P. Kubelick^2,3,4^, Jinhwan Kim^5,6^, Gabriel A. Kwong^1,2,7,8,9,10,✉^, Stanislav Y. Emelianov^1,2,3,7,8,9,10,✉^

^1^Petit Institute for Bioengineering and Bioscience, Georgia Institute of Technology, Atlanta, GA, 30332, US

^2^Wallace H. Coulter Department of Biomedical Engineering, Georgia Institute of Technology and Emory University School of Medicine, Atlanta, GA, USA

^3^School of Electrical and Computer Engineering, Georgia Institute of Technology, Atlanta, GA, USA

^4^Department of Biomedical Engineering, University of Virginia, Charlottesville, VA, USA

^5^Department of Biomedical Engineering, University of California Davis, Davis, CA, USA

^6^Department of Surgery, School of Medicine, University of California Davis, Sacramento, CA, USA

^7^Institute for Matter and Systems, Georgia Institute of Technology, Atlanta, GA, USA

^8^Cancer Technology Innovation Center, Georgia Institute of Technology,

^9^Georgia Immunoengineering Consortium, Emory University and Georgia Institute of Technology, Atlanta, GA, USA.

^10^Winship Cancer Institute, Emory University, Atlanta, GA, USA

^11^These authors contributed equally: Myeongsoo Kim, Ali Zamat

^✉^Corresponding Authors:
Gabriel A. Kwong (gkwong@gatech.edu), Stanislav Y. Emelianov ([stas@gatech.edu](mailto:stas@gatech.edu))

**Supplementary Note 1.** Experimental Details

**1.1. Fabrication of gold nanoassemblies (GNAs)**

Ellipsoidal akaganéite (β-FeOOH) nanorods with a length of 180 nm were fabricated via hydrolysis of iron chloride in an aqueous solution^[1]^. Briefly, 100 mL of 0.1 M iron chloride hydrohexahydrate (Sigma Aldrich) aqueous solution was heated up to 80 ^o^C and aged for 1 hour 40 minutes. The product was isolated by centrifugation (9,000 rpm for 15 minutes) and then redispersed in 8 mL of deionized water. To functionalize amine groups on the surface of the β-FeOOH nanorods, 8 mL of the rods were mixed with 2 mL of polyethyleneimine Sigma Aldrich, molecular weight 25,000, 40 mg/mL in deionized water), followed by shaking overnight. The amine-coated β-FeOOH nanorods were washed with deionized water via centrifugation twice (9,000 rpm for 15 minutes) and then dispersed in 10 mL of deionized water.

Gold nanospheres (2 nm) were synthesized as reported previously^[2]^. Specifically, 0.332 mL of 0.1 M sodium hydroxide aqueous solution and 8 µL of tetrakis(hydroxymethyl)phosphonium chloride solution was added into 30 mL of deionized water under magnetic stirring (1,000 rpm). To this solution, 1.92 mL of 25 mM tetrachloroauric acid trihydrate (Sigma Aldrich) aqueous solution was quickly added, followed by magnetic stirring for 1 minute. The product was aged for 24 hours at 4 ^o^C in the dark.

To conjugate 2 nm-sized gold nanospheres to the surface of amine-coated β-FeOOH nanorods, 30 mL of gold nanosphere solution was mixed with 10 mL of the β-FeOOH nanorods, followed by shaking for 30 minutes. The gold nanosphere-conjugated β-FeOOH nanorods were then isolated by centrifugation (9,000 rpm for 10 minutes) and then stabilized by 2 wt% polyvinylpyrrolidone (Sigma Aldrich, molecular weight 55,000) overnight. The stabilized constructs were washed with deionized water via centrifugation twice (9,000 rpm for 10 minutes), followed by dispersing in 13.5 mL of deionized water.

For the phenolic polymer layer (resorcinol-formaldehyde resin) coating on gold nanosphere-conjugated β-FeOOH nanorods^[3]^, 3 mL of gold-nanosphere-conjugated β-FeOOH nanorods were dispersed in 12 mL of deionized water. To this solution, 18 mg of resorcinol (Sigma Aldrich) and 25 µL of formaldehyde were added, followed by heating up to 80 ^o^C under magnetic stirring at 700 rpm. To this solution, 20 µL of ammonium hydroxide solution (Sigma Aldrich, 28 wt%) was added to the solution and the solution was heated up to 150 ^o^C. The reaction was undergone for 3.5 hours under magnetic stirring at 700 rpm, then cooling down to room temperature. The produce was washed with deionized water twice via centrifugation (9,000 rpm for 10 minutes) and dispersed in 12 mL of deionized water.

For the etching of β-FeOOH nanorods, 4 mL of gold nanosphere-conjugated β-FeOOH nanorods within the polymer layer was mixed with 4 mL of oxalic acid aqueous solution^[4]^ (Sigma Aldrich, 33.3 mg/mL), followed by incubating at 50 ^o^C overnight. The product was washed with deionized water twice via centrifugation (9,000 rpm for 10 minutes), and then dispersed in 12.5 mL of deionized water.

For the seeded growth of gold nanospheres inside the polymer layer^[5]^, 0.2 mL of gold nanospheres within the polymer layer was dispersed in 2 mL of 100 mM hexadecyl methylammonium chloride (TCI), followed by adding 0.1 mL of 25 mM tetrachloroauric acid trihydrate aqueous solution. To this solution, 0.1 mL of L-ascorbic acid (Sigma Aldrich) was added under magnetic stirring at 400 rpm. The reaction was kept for 30 minutes. It should be noted that the particle diameter of gold nanospheres was controlled by adjusting the added volume of gold precursors from 0.02 mL to 0.12 mL. The fabricated gold nanoassemblies (GNAs) were washed with deionized water twice via centrifugation (6,000 rpm for 5 minutes) and dispersed in 1 mL of deionized water.

**1.2. Characterization for GNAs**

Transmission electron microscopy (HT 7700, Hitachi) was used to examine the morphological characteristics of GNAs with different diameters of gold nanospheres. The optical spectrum for GNAs and the optical spectrum change in the seeded growth of gold nanospheres within the polymer layer were confirmed by a UV-vis-NIR spectrophotometer (Evolution 220, Theromoscientific). To characterize photoacoustic (PA) signals from GNAs, polyethylene tubes (BD Intramedic) were held in a 3D-printed scaffold. Here, the volume of GNAs were adjusted to 0.05 mL in each tube, followed by being placed in the Vevo2100/LAZR imaging system (FujiFilm VisualSonics Inc.). PA imaging data were acquired by using an LZ250 ultrasound transducer. The laser source is a Q-switched Nd:YAG-pumped optical parametric oscillator (OPO) laser (pulse duration: 7 ns, pulse rate: 20 Hz). For PA imaging, the tubes were irradiated under laser pulses at a laser fluence of approximately 10.5 mJ cm^-1^ in the 700-900 nm spectral range with 2 nm intervals. The PA gain and B-mode gain was set as 40 dB and 18 dB, respectively. To characterize photothermal responses of GNAs, GNAs were prepared in a cuvette and irradiated by an 808 nm continuous-wave (CW) laser at a laser intensity of 1 W cm^-2^ (Coherent). The temperature increase was monitored by a thermal camera (FLIR model 450sc).

The photothermal conversion efficiency (PCE) was calculated as previously reported^[6]^:

$$\eta=\frac{hS(T_{max}-T_{surr})-Q_{sys}}{I(1-{10}^{-A_{808}})}$$

where $h$is the heat transfer coefficient, $S$ is the surface area of the container exposed to the surrounding environment, $T_{\max}$ is the maximum temperature reached during laser irradiation, $T_{\mathrm{surr}}$ is the ambient temperature of the surrounding environment, $Q_{\mathrm{sys}}$ represents the heat contribution from the solvent/container system in the absence of GNAs, $I$ is the incident laser power, and $A_{808}$ is the absorbance of GNAs at 808 nm. For the calculation, the GNA suspension was irradiated with an 808 nm CW laser (1W cm^-2^), and the temperature profile was recorded until reaching a steady-state maximum temperature. The heat transfer coefficient-surface area product ($hS$) was obtained from the cooling curve after turning off the laser using the following relationship:

$$hS=\frac{\sum_{i} m_{i}C_{p,i}}{\tau_{s}}$$

where $\tau_{s}$is the system time constant obtained by fitting the cooling profile and $m_{i}C_{p,i}$ represents the product of mass and heat capacity of each component in the system. Specifically, the cooling curve was analyzed using the dimensionless temperature parameter (*θ*):

$$\theta=\frac{T-T_{surr}}{T_{max}-T_{surr}}$$

and:

$$t= -\tau_{s}ln\theta$$

The baseline heat contribution from the solvent/container system was independently measured using the same experimental setup without GNAs and calculated as:

$$Q_{\mathrm{sys}}=hS(T_{\mathrm{water}}-T_{\mathrm{surr}})$$

Using these parameters, the PCE of GNAs was calculated.

**1.3. Numerical simulation for optical responses of GNAs**

For numerical simulation, we used the finite-difference time-domain simulation software from Lumerical Inc. The surrounding temperature was set to 300 K, and the medium was water. The optical properties of gold, including dielectric constant and refractive index, were based on the values reported by Johnson and Christy^[7]^. We employed a total-field/scattered source within a spectral range of 700 nm to 900 nm. A three-dimensional model of a single GNA, incorporating varied GNS diameters, interparticle distances, and a random arrangement of GNSs based on TEM images, was used. Additionally, we set a maximum mesh step of 0.6 nm for calculating optical characteristics of the GNA. To obtain extinction, absorption, and scattering cross-sections for the GNA, we calculated the average values corresponding to light excitation at different light polarization directions, including p-polarization and s-polarization. The absorption efficiency of GNA was determined by dividing the absorption cross-section by extinction cross-section at each wavelength.

**1.4. Plasmid construction and lentivirus production**

Synthetic thermogenetic bioswitches (TS) were fabricated as previously described^[8–10]^. The NKG2DL bispecific T cell engager (TCE) sequence (US20120294858A1), previously described, was modified to include an Igκ leader sequence to facilitate secretion from CAR T cells, as well as a HisTag for construct detection^[11,12]^. This combined sequence was synthesized and then cloned downstream of the TS. VSV-G pseudotyped lentivirus was generated by transfecting HEK293 T cells (ATCC, CRL-3216) with psPAX2 (Addgene 12260) and pMD2.G (Addgene 12259). The viral supernatant was concentrated using PEG-it virus precipitation solution (System Biosciences LV825A-1) following the manufacturer’s instructions and virus was stored at -80°C until use.

**1.5. Primary human T cell production**

Peripheral blood was drawn from healthy human donors, as approved by the Georgia Tech and Emory University Institutional Review Boards (IRB #H20288). Peripheral blood mononuclear cells (PBMCs) were isolated using Lymphoprep density gradient medium (STEMCELL Technologies, 07801) and SepMate-15mL tube (STEMCELL Technologies, 85415), according to manufacturer’s instructions. CD3+ cells were then isolated using the EasySep Human CD3 Positive Selection Kit II (STEMCELL Technologies, 17851) and activated using Dynabeads (ThermoFisher, 11131D) at a 3:1 bead-to-cell ratio. Activated cells were cultured in complete human T cell media (hTCM; X- vivo 10 [Lonza #04-380Q], 5% Human AB serum [Valley Biomedical, HP1022], 10 mM N-acetyl L-Cysteine [Sigma A9165] , 55 uM 2-mercaptoethanol [Sigma, M3148-100ML] supplemented with 50 U/mL recombinant human IL-2 (TECINTM Teceleukin, Bulk Ro 23-6019, National Cancer Institute, Frederick, MD) for 24 hours at 37°C in 5% CO2. To transduce the activated human T cells, concentrated lentivirus (MOI=30) was added to a 24-well suspension culture plate coated with Retronectin (Takara, T100B) according to the manufacturer’s instructions and subsequently centrifuged at 1,200×g for 90 minutes at 37°C. Following centrifugation, activated human T cells in human T cell media supplemented with 100 units/mL of hIL-2 were added to each well and the plate was spun at 1,200×g for 60 minutes at 37°C. Cells were incubated on the virus-coated plate for 24 hours before expansion. 7 days after activation, Dynabeads were removed. For in vitro assays or in vivo therapy, transduced CAR T cells were purified using the EasySep Human CD19 Positive Selection Kit II (STEMCELL Technologies, 17854) according to the manufacturer’s protocol after 9 days of activation. All T cells were maintained at a concentration of 0.7–2×10^6^ cells/mL until Day 10-14 for use in downstream assays.

**1.6. Tumor cell culture and generation of cell lines**

Human embryonic kidney (HEK) 293T cells (ATCC) and MDA-MB-468 cells (ATCC, HTB-132) were cultured in Dulbecco’s modified Eagle medium (Gibco 11995073) supplemented with 10% FBS (Fisher 16140071) and 10 U/ml penicillin–streptomycin (Life Technologies 15140-122).

**1.7. Membrane conjugation of GNAs to human T cells**

To couple GNAs to the membrane of T cells via maleimide-thiol covalent interaction, GNAs were functionalized by maleimide moiety by a serial surface chemistry. Firstly, GNAs were coated with poly(acrylic acid) (PAA, Sigma Aldrich, molecular weight 1,800) to modify carboxylic acid. Specifically, 8 mL of GNA aqueous solution (1.54 mg mL^-1^) was mixed with 2 mL of polyvinylpyrrolidone (PVP, Sigma Aldrich, molecular weight 55,000) aqueous solution, followed by shaking overnight and washing with centrifugation twice (9,000 rpm for 10 minutes). The PVP-coated GNAs were then dispersed in 8 mL of deionized water and mixed with 2 mL of PAA aqueous solution (40 mg mL^-1^), followed by sonication for 2 hours. The PAA-coated GNAs were washed with deionized water via centrifugation three times (9,000 rpm for 10 minutes) and then dispersed in 4 mL of deionized water. Next, the PAA-coated GNAs were mixed with 0.2 mL of 20 mM 1-(3-dimethylaminopropyl)-3-ethylcarbodiimide (EDC, Alfa Aesar) and 0.2 mL of 20 mM N-hydroxysulfossuccinimide sodium salt (sulfo-NHS, bioWORLD), followed by shaking for 1 hour. The EDC/NHS-activated GNAs were washed with deionized water three times via centrifugation (9,000 rpm for 5 minutes). To this solution, 0.2 mL of 1 mM NH_2_-PEG-NH_2_ (BroadPharm, molecular weight 2,000) and 1.8 mL of 1 mM mPEG-NH_2_ (Creative PEGWorks, molecular weight 2,000) were added, followed by shaking overnight. The PEGylated GNAs were washed with deionized water three times via centrifugation (9,000 rpm for 5 minutes) and dispersed in 4 mL of deionized water. For maleimide (MAL) functionalization, 4 mL of PEGylated GNAs (3.08 mg mL^-1^) were mixed with 0.4 mL of 1 mM MAL-PEG_4_-NHS ester (BroadPharm, molecular weight 442.4) and then shaken for 4 hours. The product was washed with phosphate buffer saline (Corning, PBS) three times via centrifugation (12,000 rpm for 3 minutes) and then dispersed in 4 mL of PBS solution. The surface functionalization process was characterized by using a dynamic light scattering instrument (Zetasizer Nano ZS, Malvern Instruments Ltd.)

To conjugate maleimide-functionalized GNAs to the membrane of human T cells, the cells were re-dispersed in PBS solution at a cell density of 5 million cells mL^-1^. GNA solution with varying doses per T cell (from 0 to 308 picogram cell^-1^) was added to the cell solution, followed by incubating for 30 minutes. To block remaining maleimide groups on GNAs, we added 1 mL of 1 mM mPEG-SH (Biochempeg, molecular weight 2,000) to the GNA-containing cell solution, incubated another 10 minutes, and then washed with human T cell media three times via centrifugation (600×g for 3 minutes). The GNA-bound T cells were dispersed in the human T cell media for further use.

**1.8. Scanning electron microscopy (SEM) for GNA-bound T cells**

For SEM imaging of GNA-bound T cells, the cells were fixed by treating 4% paraformaldehyde solution for 15 minutes, followed by isolation via centrifugation (600×g for 3 minutes). The cells were dehydrated by incubating with 10%, 30%, 50%, 70%, 90%, and 100% ethanol (10 minutes for each step), pipetted onto a small piece of silicon wafer (Electron Microscopy Sciences, Type P, 6”), and then airdried. The dried samples were then coated with ~ 10 nm gold-palladium layer for SEM imaging (SU8230, Hitachi).

**1.9. Staining and flow cytometry**

NIR Live/Dead (ThermoFisher L34976) and CellTrace Violet (LifeTech C34557) were used following the manufacturer’s instructions.  Prior to antibody staining, human Fc block (BD 564220) was applied. Antibodies for CD25 (M-A251; BioLegend #356104), CD69 (FN50; BioLegend #310930), HER2 (24D2; BioLegend #324404), β2-microglobulin (A17082A, BioLegend #395704), CD3 (SK7; BioLegend #344822), CD4 (OKT4, BioLegend #317444), CD8 (SK1, BioLegend #344704), CD19 (HIB19, BioLegend #302212), His-Tag for TCE (AD1.1.10, R&D Systems #IC050A), Ki67 (11F6, BioLegend #151212), and IFN-γ (4S.B3, BioLegend #502540) were all used at 1:100 dilutions. For staining exofacial thiol groups on WT T cells, Maleimide-PEG-FITC (1845706-65-1, AA Blocks) was utilized. For immunohistochemistry analysis, anti-CD3ε antibody (EP449E, abcam) was utilized.

**1.10. Viability, proliferation, migration, and T cell activation assays**

Viability, proliferation, migration, and T cell activation were assessed on primary human T cells with varying GNA doses. Viability was quantified immediately after membrane conjugation of GNAs, 24 hours post labeling, after imaging, and heating using LIVE/DEAD Fixable Near-IR Dead Cell Stain Kit (Thermofisher Scientific L34975) and evaluated by flow cytometry. Specifically, the effects of membrane-bound GNAs, pulsed laser irradiation, and CW laser irradiation on T cell viability were evaluated by calculating the percentage of viable cells in each experimental group relative to the corresponding control group (i.e., unconjugated T cells for GNA conjugation experiments and untreated T cells for irradiation experiments), with each control group normalized to 100%. To evaluate T cell proliferation following particle labeling, T cells were first labeled with CellTrace^TM^ Violet (CTV) Cell Proliferation Kit (Thermofisher C34557) prior to GNA labeling. Briefly, cells were stained in 2.5µM PBS solution at 37 ^o^C in the dark for 10 minutes. Afterward, 5 volumes of complete human T cell media were added, and cells allowed to incubate for 5 more minutes at 37 ^o^C. After labeling, cells were conjugated to GNAs and then activated using Dynabeads (ThermoFisher, 11131D) at a 1:1 bead-to-cell ratio and incubated for 48 hours. Proliferation and division indices were calculated in FlowJo using the proliferation tool. For migration studies, GNA-conjugated WT T cells or unconjugated WT T cells were added to the top chamber of transwell plate (Sigma Aldrich CL3421), while CXCL12 (50 ng mL^-1^, Reprotech 300-28 A) was added to the lower chamber. Following an incubation period of 18 hours, the cells in the lower chamber were quantified through manual counting. T cell activation was assessed by quantifying CD69 and CD25 levels on the surface of T cells. Briefly, T cells were GNA-conjugated and activated using Dynabeads (ThermoFisher, 11131D) at a 1:1 bead-to-cell ratio and incubated for 48 hours before the flow cytometric analysis.

**1.11. In vitro US/PA imaging of GNA-WT T cells**

GNA-bound WT T cells (GNA-WT T cells) were dispersed in PBS solution at a cell density of 800, 400, 200, 100 or 0 cells µL^-1^. Next, to prepare tissue-mimicking dome-phantoms^[13]^, 15 µL of the PBS solution containing GNA-WT T cells was mixed with 15 µL of 16% gelatin (MP Biomedicals, LLC) solution. The mixture was pipetted to create a dome shape on a gelatin/silica phantom base. The domes were solidified for 4 hours at 4 ^o^C before PA imaging. For PA imaging of the domes containing GNA-WT T cells, the phantom base was placed in a distilled water-filled container in the Vevo2100/LAZR imaging system. The domes were imaged in the 700-900 nm spectral region with 2 nm intervals. The PA gain and B-mode gain were set as 40 dB and 18 dB, respectively. For photostability test of GNA-WT T cells, we acquired PA signals from domes at 800 nm for 1,000 pulses with fluence of 10.5 mJ cm^-2^.

**1.12. Cytotoxicity assays**

Wildtype human T cells (WT T cells) or αHER2 CAR T cells were prepared and underwent surface coupling with GNAs at a dose of 0 pg cell^-1^ or 154 pg cell^-1^. Next, WT T cells or αHER2 CAR T cells uncoupled or coupled with GNAs were co-incubated with HER2+ and HER2- MDA-MB-468 cells at a 2:1 effector to target ratio. Cytotoxicity of these cells against HER2+ or HER2- MDA-MB 468 cells were recorded as a measure of cell impedance using the xCELLigence RTCA eSight instrument (Agilent Technologies).

**1.13. Adoptive cell transfer and in vivo US/PA imaging of the trafficking of GNA-αHER2 CAR T cells to MDA-MB 468 tumors with different HER2 expression levels**

The ACT experiments with in vivo US/PA imaging of trafficking of GNA-αHER2 CAR T cells to MDA-MB 468 tumors with different HER2 expression levels were performed under the Institutional Animal Care and Use Committee (IACUC, protocol number: A100281) guidelines of the Georgia Institute of Technology. NSG mice were inoculated with 5x10^6^ MDA-MB-468 tumor cells comprised of a 0%, 10%, 50%, or 100% HER2+ mixture as determined by flow cytometry and resuspended in 100 μL PBS-/- injected into the left flank. GNA-αHER2 CAR T cells (5 million cells in 0.2 mL of saline solution) were intravenously injected. For imaging studies, mice were anesthetized using inhaled isoflurane mixed with 100% oxygen. Isoflurane concentrations ranged from 1.5% to 2.0%, delivered at an oxygen flow rate of 0.4-0.8 L min^-1^. In vivo US/PA images were acquired using the Vevo LAZR 2100 imaging system, employing LZ250 transducers. The laser source employed was a Q-switched Nd:YAG pumped OPO laser, with a pulse repetition frequency of 20 Hz and a pulse duration of 7 nanoseconds. All acquired data were analyzed using VevoLAB5.7.0 and MATLAB R2023a software.

**1.14. In vitro photothermal activation of GNA-TS-Gluc T cells or GNA-TS-TCE αHER2 CAR T cells.**

To explore the impact of membrane-bound GNAs on the thermal activation of TS-engineered T cells, Gluc expression (Gaussia Luciferase Glow Assay Kit, ThermoFisher 16161) from both GNA-bound TS-Gluc T cells and unbound TS-Gluc T cells was quantified by using an IVIS Spectrum CT (PerkinElmer) following laser irradiations for 1,000 pulses at a laser fluence of 10.5 mJ cm^-2^, thermocycler- or CW laser-mediated heat treatment at 42 ^o^C for 20 minutes. The heating process was carried out by using an 808 nm continuous wave (CW) laser at a laser intensity of 1 W cm^-2^ (Coherent). The temperature increase was monitored by a thermal camera (FLIR model 450sc). To investigate the potential of thermal activation to trigger expression of an NKG2D ligand-targeted TCE on CAR T cells coupled with GNAs, the cytotoxicity of GNA-TS-TCE αHER2 CAR T cells was assessed against HER2+ or HER2- MDA-MB-468 cells using the xCELLigence RTCA eSight instrument (Agilent Technologies) before and after heat treatment at 42 ^o^C for 20 minutes, while comparing this with the killing activity of GNA-WT T cells.

**1.15. ACT and photothermal activation of intratumoral GNA-TS-TCE αHER2 CAR T cells**

The ACT experiments with localized thermal activation of TCE expression on GNA-bound CAR T cells engineered with thermogenetic switch were performed under the Institutional Animal Care and Use Committee (IACUC, protocol number: A100191) guidelines of the Georgia Institute of Technology. NSG mice were inoculated with 5x10^6^ MDA-MB-468 tumor cells comprised of a 10% HER2+ mixture and resuspended in 100 μL PBS-/- injected into the left flank. GNA-TS-TCE αHER2 CAR T cells, GNA-TS-Fluc αHER2 CAR T cells, GNA-WT T cells, or WT T cells (5 million cells in 200 μL PBS-/-) were intravenously injected by tail vein. For PT heating studies, mice were anesthetized using inhaled isoflurane mixed with 100% oxygen. Isoflurane concentrations ranged from 1.5% to 2.0%, delivered at an oxygen flow rate of 0.4-0.8 L min^-1^. Photothermal heating of intratumoral CAR T cells (42 ^o^C for 10 minutes, 5 minutes of rest followed by 42 ^o^C for another 10 minutes) was conducted by using an 808 nm continuous wave (CW) laser with controlled laser intensity (Coherent) to maintain a target temperature of 42 ^o^C. The laser intensity was initially set to approximately 1 W cm^-2^ and subsequently modulated to sustain this temperature. The surface temperature of tumor during irradiation was monitored by a thermal camera (FLIR model 450sc). To evaluate tumor composition and quantify T cell counts, tumors were enzymatically and mechanically dissociated using the Human Tumor Dissociation Kit (Miltenyi, 130-095-929) and gentleMACS Dissociator (Miltenyi, 130-093-235) as per manufacturer’s instructions. Cells were then isolated from a single cell suspension using a density gradient with Percoll Centrifugation Media (VWR, 17-5445-01) and RPMI Media (Cytiva, SH30027.FS) at a 44:56 volume ratio before staining and flow cytometric analysis.

**1.16. Software and statistical analysis**

Statistical analysis was performed using GraphPad Prism 8.0.2 statistical software. Flow cytometry data were collected with Aurora (Cytek) and analyzed using FlowJo X (FlowJo). All acquired US/PA imaging data were analyzed using VevoLAB5.7.0 and MATLAB software. In vitro IVIS data were collected and analyzed with Living Image 4.4.5 (PerkinElmer). Figures were designed in Adobe illustrator. No data were excluded from the analyses.

**Supplementary Note 2.** Supplementary Figures

**Figure S1 | a,** Schematic illustration of fabrication of GNAs. **b-f,** TEM images of FeOOH nanorods (**b**), gold seed-coated FeOOH nanorods (**c**), gold seed-coated FeOOH nanorods within a polymer layer (**d**), gold seeds inside the polymer layer (**e**), and GNAs (**f**). The scale bars are 200 nm.

**Figure S2| a,** TEM images of GNAs with tunable diameter and interparticle spacing of GNSs by modulating the added volume of gold ions in the seeded growth process. The scale bars are 200 nm. **b-g** UV-vis-NIR spectra (**b**), PA signal generation (**c-f**, n=4), and heat generation (**g**, n=3) from GNAs with different diameters and interparticle spacing of GNSs. Data represents the mean ± s.d. The statistical analysis was conducted through one-way analysis of variance (ANOVA) using Tukey post-hoc test. The statistically non-significant differences are represented as ns.

**Figure S3 |** Calculated extinction, absorption, and scattering cross-sections for GNAs in the 700-900 nm spectral range.

**Figure S4 |** Calculation of the photothermal conversion efficiency of GNAs at a wavelength of 808 nm. **a**, Temperature profile of the GNA solution under 808 nm CW laser irradiation (1 W cm^-2^). **b**, Determination of the system time constant from the cooling period.

**Figure S5 |** TEM images of GNRs or GNAs after CW laser illumination (1 W cm^-2^ for 5 minutes). The scale bars are 200 nm.

**Figure S6 |** UV-vis-NIR spectra of GNAs after pulsed laser irradiations for 1,000 pulses at different laser fluences.

**Figure S7 |** UV-Vis-NIR spectra (a) and hydrodynamic diameters (b, n=3) of GNAs over 20 days. Data presents mean ± s.d.

**Figure S8 |** Exofacial thiol flow staining of WT T cells. SSC-A, side scatter area.

**Figure S9 | a,** Schematic illustration of the serial surface chemistry for the maleimide functionalization on GNAs for the membrane conjugation to T cells. **b,** Corresponding surface charge changes of the GNAs in the serial surface functionalization (n=3), mean ± s.d.

**Figure S10 |** Ratio of CD4 to CD8 in T cells before and after membrane conjugation of GNAs (n=3). Data presents mean ± s.e.m.

**Figure S11 | a**, Schematic illustration of membrane conjugation of GNAs to primary human T cells via maleimide-thiol coupling. **b, c** Low magnification of SEM images of WT T cells before (**b**) and after the conjugation of GNAs (**c**). Scale bars are 5 µm.

**Figure S12 |** Migration of T cells in the lower well of a transwell plate with or without the addition of CXCL12 after 18 hours (n=3), mean ± s.e.m. The statistical analysis was conducted using an unpaired two-tailed t-test.

**Figure S13 | a,** SEM images of GNA-WT T cells at day 0 and day 2, respectively. The scale bars are 2 µm. **b,** Surface GNA densities on T cells at day 0 and day 2, respectively (n=18). **c,** total number of T cells at day 0 and day 2, respectively (n=3). The statistical analysis was conducted using an unpaired two-tailed t-test.

**Figure S14 | a-c** PA signal generation from GNA-WT T cells (coupled with different GNA doses) at the wavelength of 700 nm, 800 nm, and 900 nm (n=3), mean ± s.e.m. ns = non-significant, *P < 0.05, **P < 0.01, *** P < 0.001, ****P < 0.0001; one-way ANOVA with Tukey post-hoc test.

**Figure S15 | a, b** Cell viability quantified by flow cytometry following PA imaging protocol for 1,000 laser pulses, 250 imaging frames (n=3), mean ± s.e.m. The statistical analysis was conducted using an unpaired two-tailed t-test. The statistically non-significant difference is represented as ns.

**Figure S16 |** Cytotoxicity of αHER2 CAR T cells or WT T cells coincubated with MDA-MB-468 tumor cells after GNA conjugation at an effector-to-target ratio of 2:1 (n=3), mean ± s.e.m

**Figure S17 |** HER2 flow staining of MDA-MB 468 cells. FSC-A, forward scatter area.

**Figure S18 |** US/PA imaging (800 nm) of tumors with varying HER2 expression at indicated time points post-ACT. The scale bars are 4 mm.

**Figure S19 |** Individual spider plots of PA amplitude (800 nm) of tumors with varying HER2 expression at indicated time points post-ACT.

**Figure S20 |** Individual spider plots of relative tumor volume post-ACT.

**Figure S21 |** Relative change in tumor burden (volume) across ACT-treated mice with varying HER2 compositions (n=19), including responders (n=9) and nonresponders (n=10).

**Figure S22** | **a, b** Representative images of 0% HER2+ (**a**) or 100% HER2+ (**b**) tumors stained with H&E and immunostained for CAR T cells (Fast Red chromogen, red/pink signal) at Day 4 post-ACT. **c**, Number of T cells per tumor volume in 0% or 100% HER2+ tumors at Day 4 post-ACT (n=5). Data presents mean ± s.e.m., *P < 0.05: an unpaired two-tailed t-test.

**Figure S23** | Percentage of CD25+CD69+ in T cells in 0% (nonresponder) or 100% HER2+ MDA-MB-468 tumors (responder) at Day 4 post-ACT (n=5). Data presents mean ± s.e.m., *P < 0.05: an unpaired two-tailed t-test.

**Figure S24 | a, b** Representative IVIS image and quantification of Gluc expression from TS-Gluc T cells quantified in supernatant following thermocycler-mediated heating for 20 minutes at 42 ^o^C with or without GNA membrane conjugation (n=3), mean ± s.e.m. The statistical analysis was conducted using an unpaired two-tailed t-test. The statistically non-significant difference is represented as ns.

**Figure S25 |** Cell viability quantified by flow cytometry following CW laser-mediated heating protocol (n=3), mean ± s.e.m. The statistical analysis was conducted using an unpaired two-tailed t-test. The statistically non-significant difference is represented as ns.

**Figure S26 |** Percentage of TCE+ cells among WT, GNA-WT, TS-TCE αHER2 CAR T, and GNA-TS-TCE αHER2 CAR T cells with or without heat treatment at 42 °C for 20 min (n=3). Data presents mean ± s.e.m.

**Figure S27 |** Tissue thickness-dependent laser-mediated heating of GNA solutions through chicken breast layers under 808 nm CW laser irradiation at a laser intensity of 1 W cm^-2^ (n=3). Data presents mean ± s.d.

**Figure S28 |** Repeated heat treatment does not impair T cell viability, proliferation, or activation. **a**, Schematic of the treatment and analysis timeline. GNA-bound or unbound WT T cells were unstimulated or stimulated with dynabeads on day 1, and/or subjected to mild heat treatment (42°C for 20 minutes) on days 1, 3, and 5, followed by flow cytometry on day 6. **b**, T cell viability across the four treatment groups, with or without membrane-bound GNAs. **c**, Frequency of Ki67+IFN-γ+ cells among CD3+ T cells. **d**, CD69 expression among CD3+ T cells. Data presents mean ± s.e.m.

**Figure S29 |** Quantifications of tumor burden post-ACT (n=5, mean ± s.e.m.). Laser treatments were only applied to tumors treated with GNA-WT T cells. Each cohort intravenously received 5M of cells in 200 µL of saline solution. The statistical analysis was conducted using an unpaired two-tailed t-test. The statistically non-significant difference is represented as ns.

**Figure S30 |** HER2 flow staining of MDA-MB-468 cells. FSC-A, forward scatter area.

**Figure S31 |** Individual spider plots of tumor volumes post-ACT.

**Figure S32 |** Number of HER2-negative tumor cells in tumors on Day 38 post-ACT (n = 4 to 6), mean ± s.e.m., ns = non-significant, *P < 0.05, **P < 0.01; one-way ANOVA with Tukey post-hoc test.

**References**

[1]	Y. Piao, J. Kim, H. B. Na, D. Kim, J. S. Baek, M. K. Ko, J. H. Lee, M. Shokouhimehr, T. Hyeon, *Nat. Mater.* **2008**, *7*, 242.

[2]	J. Kim, D. Seo, J. Lee, K. M. Southard, Y. Lim, D. Kim, Z. J. Gartner, Y. Jun, J. Cheon, *Nat. Protoc.* **2017**, *12*, 1871.

[3]	N. Li, Q. Zhang, J. Liu, J. Joo, A. Lee, Y. Gan, Y. Yin, *Chem. Commun.* **2013**, *49*, 5135.

[4]	S. O. Lee, T. Tran, B. H. Jung, S. J. Kim, M. J. Kim, *Hydrometallurgy* **2007**, *87*, 91.

[5]	M. Kim, J. Kim, D. VanderLaan, K. P. Kubelick, A. Jhunjhunwala, A. Choe, S. Y. Emelianov, *Adv. Funct. Mater.* **2023**, *33*, 2305202.

[6]	J. R. Cole, N. A. Mirin, M. W. Knight, G. P. Goodrich, N. J. Halas, *J. Phys. Chem. C* **2009**, *113*, 12090.

[7]	P. B. Johnson, R. W. Christy, *Phys. Rev. B* **1972**, *6*, 4370.

[8]	M. Gaestel, R. Gotthardt, T. Müller, *Gene* **1993**, *128*, 279.

[9]	S. J. Priceman, D. Tilakawardane, B. Jeang, B. Aguilar, J. P. Murad, A. K. Park, W.-C. Chang, J. R. Ostberg, J. Neman, R. Jandial, J. Portnow, S. J. Forman, C. E. Brown, *Clin. Cancer Res.* **2018**, *24*, 95.

[10]	L. N. Hwang, Z. Yu, D. C. Palmer, N. P. Restifo, *Cancer Res.* **2006**, *66*, 1132.

[11]	I. C. Miller, A. Zamat, L.-K. Sun, H. Phuengkham, A. M. Harris, L. Gamboa, J. Yang, J. P. Murad, S. J. Priceman, G. A. Kwong, *Nat. Biomed. Eng.* **2021**, *5*, 1348.

[12]	I. C. Miller, M. Gamboa Castro, J. Maenza, J. P. Weis, G. A. Kwong, *ACS Synth. Biol.* **2018**, *7*, 1167.

[13]	J. R. Cook, R. R. Bouchard, S. Y. Emelianov, *Biomed. Opt. Express* **2011**, *2*, 3193.
